# Intracranial pressure variability: relation to clinical outcome, intracranial pressure–volume index, cerebrovascular reactivity and blood pressure variability

**DOI:** 10.1007/s10877-019-00387-9

**Published:** 2019-09-19

**Authors:** Teodor Svedung Wettervik, Timothy Howells, Per Enblad, Anders Lewén

**Affiliations:** Section of Neurosurgery, Department of Neuroscience, Uppsala University, 751 85 Uppsala, Sweden

**Keywords:** Traumatic brain injury, Intracranial pressure variability, Neurointensive care, Clinical outcome

## Abstract

It was recently found in traumatic brain injury (TBI) that ICP variability (ICPV) predicted favorable outcome. We hypothesized that ICPV may depend on intracranial compliance, unstable blood pressure and cerebral vasomotion. In this study, we aimed to further investigate the explanatory variables for ICPV and its relation to outcome. Data from 362 TBI patients were retrospectively analyzed day 2 to 5 post-injury. ICPV was evaluated in three ways. First, variability in the sub-minute time interval (similar to B waves) was calculated as the amplitude of the ICP slow waves using a bandpass filter, limiting the analysis to oscillations of 55 to 15 s (ICP AMP 55–15). The second and third ICPV measures were calculated as the deviation from the mean ICP averaged over 30 min (ICPV-30m) and 4 h (ICPV-4h), respectively. All ICPV measures were associated with a reduced intracranial pressure/volume state (high ICP and RAP) and high blood pressure variability in multiple linear regression analyses. Higher ICPV was associated with better pressure reactivity in the univariate, but not the multiple analyses. All ICPV measures were associated with favorable outcome in univariate analysis, but only ICP AMP 55–15 and ICPV-30m did so in the multiple logistic regression analysis. Higher ICPV can be explained by a reduced intracranial compliance and variations in cerebral blood volume due to the vessel response to unstable blood pressure. As ICP AMP 55–15 and ICPV-30m independently predicted favorable outcome, it may represent general cerebral vessel activity, associated with better cerebral blood flow regulation and less secondary insults.

## Introduction

Traumatic brain injury (TBI) is a leading cause of morbidity and mortality in young adults worldwide [1]. Post-traumatic intracranial hypertension is caused by expanding intracranial hemorrhages and cerebral edema and is associated with increased mortality [2]. However, although intracranial hypertension is associated with reduced intracranial compensatory reserve, we and others have found that intracranial pressure (ICP) variability is associated with favorable outcome [3–5].

ICP variability (ICPV) can be defined over various time intervals. The slow wave ICP amplitude, i.e. ICP oscillations with time periods at e.g. 15 to 55 s, is similar to “B waves” and is believed to represent the vasogenic response to blood pressure variations [3]. ICPV may also be calculated over longer time intervals as the standard or mean absolute deviation from a mean ICP averaged for a defined time interval such as hours or days, but it is less clear what these variabilities represent [4].

The exact physiological mechanisms for ICP variability in short and long-term time intervals, their physiological information and relation to outcome are poorly studied. In this study, we aimed to investigate the differences in very short-term (sub-minute window), short-term (minutes) and long-term (hours) ICP variability, its relation to ICP and other physiological variables to determine how this information is associated with clinical outcome.

## Materials and methods

### Study design and participants

The Department of Neurosurgery at the University Hospital in Uppsala, Sweden, provides neurosurgical care for a central part of Sweden, with a population of approximately two million people. Most patients are initially managed at local hospitals according to the advanced trauma life support (ATLS) principles and then referred to Uppsala (the most distant hospital 382 km away) [6]. Since 2008, all patients with TBI admitted to our neurointensive-care (NIC) unit, are included in the Uppsala Traumatic Brain Injury web based (TBI) register [7].

### Treatment protocol

All patients were treated in accordance with a standardized ICP-oriented treatment protocol to avoid secondary insults [8, 9]. Treatment goals were ICP ≤ 20 mmHg, CPP ≥ 60 mmHg, systolic blood pressure > 100 mmHg, central venous pressure 0–5 mmHg, pO_2_ > 12 kPa, blood glucose 5–10 mmol/L, electrolytes within normal ranges together with normovolemia and body temperature < 38 °C.

All unconscious (GCS M 1-5) patients were intubated and sedated with propofol infusion (Propofol-LipuroB, Braun Medical, Danderyd, Sweden) and morphine (Morfin Media; Media, Sollentuna, Sweden) for analgesia. The intracranial pressure was monitored in all unconscious patients with either an intraparenchymal sensor device (Codman ICP Micro-Sensor, Codman & Shurtleff, Raynham, MA) or an intraventricular catheter drainage system (HanniSet, Xtrans, Smith Medical GmbH, Glasbrunn, Germany). Patients were initially hyperventilated (4.0–4.5 kPa), but normoventilated as soon as ICP allowed. In stable patients, neurological wake-up tests were repeatedly performed. In case of high ICP with simultaneous high blood pressure and tachycardia, stress was treated with a β_1_-antagonist infusion (Seloken; AstraZeneca, Södertälje, Sweden) and repeated injections of a α_2_-agonist (Catapresan; Boehinger Ingelheim, Stockholm, Sweden).

Intracranial lesions with significant mass effect were surgically evacuated. In situations of increased ICP, despite basal treatment and if no mass lesion was present, cerebrospinal fluid was drained. If ICP still remained elevated, a thiopental infusion was started, and finally, if high ICP was still refractory, a decompressive craniectomy was performed.

### Physiological analysis

**Fig. 1:**
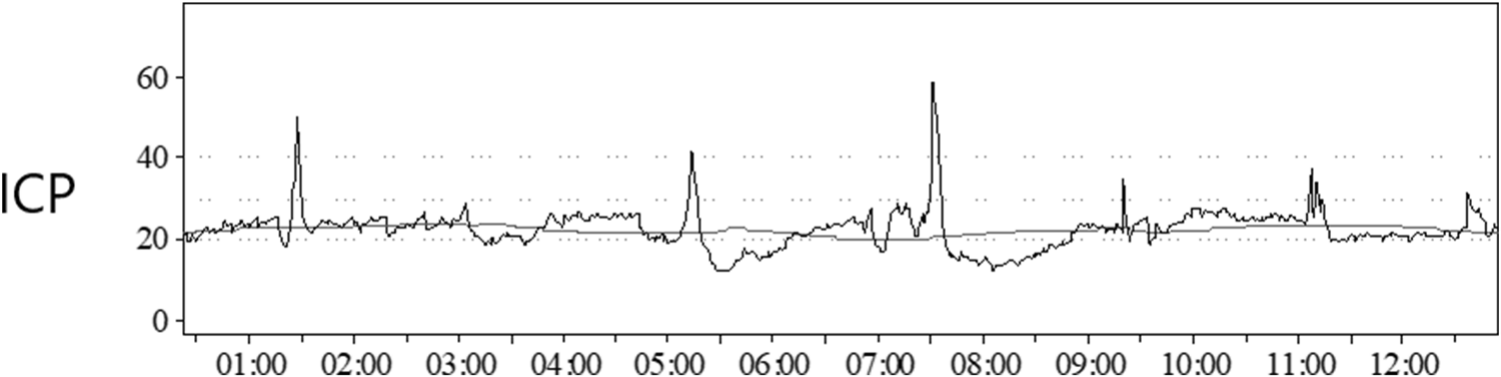
Calculation of the ICPV-4h. The figure demonstrates an ICP curve (several spikes) together with the 4-h moving average (flat, no spikes). The mean absolute deviation of the ICP curve from the moving 4-h-average ICP was calculated as ICPV-4h (values not shown in figure)

The ICP and arterial blood pressure data were recorded with the Odin software, developed at Uppsala University and University of Edinburgh [10]. ICPV was analyzed in three ways with different time intervals. First, in the sub-minute window, the slow wave ICP amplitude 55–15 (ICP AMP 55–15) was calculated as the ICP amplitude of ICP waves with a bandpass filter generated by the Odin Software, limiting the analysis to ICP oscillations with periods 55 to 15 s. The second and third ICPV measures, i.e. ICPV-30m and ICPV-4h, were computed for every minute of monitoring as the absolute deviation from a 30-min and 4-h moving average centered on the minute, respectively. An example of the ICPV-4h calculation is demonstrated in Fig. 1. The temporal trends for these three ICPV measures were evaluated the first 10 days post-injury for those with favorable and unfavorable outcome. We then chose to focus on the physiological data day 2 to 5, when the physiological data have been found to have the highest outcome prediction [5].

PRx, which originally was described by Czosnyka et al., was calculated as a moving 5-min correlation of 10 s averages of ICP and MAP [11]. We also used a variant of PRx, PRx55–15, that was calculated in a similar way as PRx, but with a bandpass filter limiting the analysis to oscillations with periods from 55 to 15 s [5, 12].

The RAP-index (R, amplitude and pressure compliance index) was calculated as the moving 5-min correlation between ICP amplitude and ICP [13]. ART AMP 55–15 was calculated as the blood pressure amplitude of blood pressure waves with a similar bandpass filter, limiting the analysis to oscillations with periods from 55 to 15 s, similar to ICP AMP 55–15. Blood pressure variability (BPV) was also calculated as BPV-30m and BPV-4h, i.e. computed for every minute of monitoring as the absolute deviation from a 30-min and 4-h moving average centered on the minute, respectively.

### Outcome

Outcome was assessed at 6 months following injury, by specially trained personnel with structured telephone interviews, using the Extended Glasgow Outcome Scale (GOS-E) [14, 15], containing eight categories of global outcome, from death (GOS-E 1) to upper good recovery (GOS-E 8). GOS-E scores of 1 to 4 were considered unfavorable outcome, and 5 to 8 favorable.

### Statistical methods

Demographic data were presented as the mean ± SD. Mean daily values for all three ICPV measures were generated for each patient the first 10 days in Odin, as illustrated in Fig. 3. As day 2 to 5 was the most sensitive time interval for the ICPV measures for outcome prediction [5], mean values for this 96-h-period were calculated of ICP, RAP, CPP, PRx, PRx55–15, ART Amp 55–15, BPV-30m, BPV-4h, ICP Amp 55–15, ICPV-30m and ICPV-4h in the Odin software. We also calculated the good monitoring time (GMT) % day 2 to 5 of CPP in the optimal range 60 to 70 mmHg according to the Brain Trauma foundation [16] and ICP > 20 mmHg. All of these measures were based on minute-by-minute data. Physiological and demographic data were transferred to SPSS version 25 (IBM Corp, Armonk, NY, USA).

The explanatory variables for the three ICPV measures were analyzed with univariate correlation tests (Spearman) and multiple linear regression including demographic data (age, GCS M, pupillary status and CT Marshall score) and physiological data (ICP, CPP, PRx and ART Amp 55–15/BPV-30m/BPV-4h) for day 2 to 5 (Table 1). As PRx had a stronger correlation with the ICPV measures in the univariate analyses it was preferred instead of PRx55–15 as the independent variable in the multiple linear regression analyses.

**Table 1:** ICP variability in relation to demographic and neurophysiological parameters—univariate (Spearman rank correlation) and multiple linear regression analyses

	ICP AMP 55–15				ICPV-30m				ICP-4h			
	Spearman		Multiple linear regression		Spearman		Multiple linear regression		Spearman		Multiple linear regression	
	r	p value	SC	p value	r	p value	SC	p value	r	p value	SC	p value
Age	0.001	0.98	− 0.11	0.03	− 0.17	0.002	− 0.10	0.10	− 0.20	< 0.001	− 0.16	0.01
GCS M	0.15	0.005	0.03	0.45	0.17	0.003	0.03	0.54	0.14	0.01	0.02	0.71
Pupils	− 0.21	< 0.001	0.09	0.05	− 0.22	< 0.001	− 0.11	0.04	− 0.19	< 0.001	− 0.09	0.10
Marshall	− 0.13	0.018	− 0.05	0.24	− 0.21	< 0.001	− 0.06	0.30	− 0.20	< 0.001	− 0.03	0.65
ICP	0.23	< 0.001	0.23	< 0.001	0.22	< 0.001	0.22	< 0.001	0.19	< 0.001	0.22	< 0.001
RAP	0.59	< 0.001	0.51	< 0.001	0.49	< 0.001	0.27	< 0.001	0.44	< 0.001	0.20	0.001
MAP	0.092	0.09	0.002	0.97	− 0.07	0.21	− 0.13	0.021	− 0.12	0.03	− 0.27	< 0.001
ART AMP 55–15	0.27	< 0.001	0.38	< 0.001	NA	NA	NA	NA	NA	NA	NA	NA
BPV-30m	NA	NA	NA	NA	0.11	0.049	0.18	0.002	NA	NA	NA	NA
BPV-4h	NA	NA	NA	NA	NA	NA	NA	NA	− 0.02	0.66	0.22	< 0.001
PRx55–15	− 0.08	0.15	NA	NA	− 0.08	0.15	NA	NA	− 0.10	0.09	NA	NA
PRx	− 0.15	0.005	− 0.03	0.55	− 0.18	0.001	0.02	0.80	− 0.15	0.005	0.06	0.34
DC	− 0.32	< 0.001	− 0.15	< 0.001	− 0.28	< 0.001	− 0.16	0.003	− 0.19	< 0.001	− 0.14	0.009

*SC* standardized coefficient, *NA* not applicable

Pupils (0 = normal, 1 = abnormal). DC (0 = no, 1 = yes). Regressions: ICP AMP 55–15, R^2^ = 0.52, ANOVA, p value < 0.001. ICPV-30m, R^2^ = 0.25, ANOVA, p value < 0.001. ICPV-4h, R^2^ = 0.21, ANOVA p value < 0.001

Each of the three ICPV measures was evaluated for association with outcome (favorable/unfavorable) with simple and multiple logistic regression analyses (Table 2). The multiple logistic regression analyses included, in addition to the ICPV measure, demographic data (age, GCS M, pupillary status) and physiological data (GMT  % of ICP > 20 mmHg, GMT % of 70 mmHg > CPP > 60 mmHg, PRx55–15 and ART Amp 55–15/BPV-30m/BPV-4h) for day 2 to 5. Decompressive craniectomy (DC, yes/no) was also included as an independent variable, to adjust for possible effects on the neurophysiological parameters without an intact skull. The difference in ICPV between favorable and unfavorable outcome was also demonstrated with *t* tests.

**Table 2:** ICPV and prediction of unfavorable outcome—simple and multiple logistic regression analyses

	Simple		Multiple	
	OR (95% CI)	p value	OR (95% CI)	p value
ICP AMP 55–15	0.71 (0.56–0.89)	0.003	0.73 (0.54–0.99)	0.047
ICPV-30m	0.23 (0.12–0.45)	< 0.001	0.43 (0.20–0.96)	0.04
ICPV-4h	0.54 (0.34–0.84)	0.007	0.90 (0.57–1.4)	0.90

Three simple logistic regression were performed for each ICPV measure. Furthermore, three multiple logistic regression analyses were performed to evaluate if each of the three ICPV measures independently predicted clinical outcome. Age, GCS M, pupillary status, decompressive craniectomy, ICP, CPP, PRx55–15 and blood pressure variability were included as independent variables in addition to the ICPV measure in each multiple regression. The blood pressure variability with the corresponding time interval as the ICPV was included in each analysis, e.g. ICP AMP 55–15 and ART AMP 55–15

*OR* odds ratio, *CI* confidence interval

Furthermore, the ICPV measures were evaluated for association with intracranial hypertension. The mean ICPV measures for the first day post-injury and the proportion of good monitoring time (GMT) (%) of ICP > 20 mmHg were calculated for day 1 and day 2 to 5, respectively. The correlation between the ICPV measures on day 1 vs. GMT > 20 mmHg on day 1 and day 2 to 5 were analyzed with the Spearman correlation test (Table 3).

**Table 3:** Early ICP variability and prediction of intracranial hypertension (Spearman)

	GMT ICP > 20 day 1 (%)		GMT ICP > 20 day 2 to 5 (%)	
	r	p value	r	p value
ICP AMP 55–15 day 1	0.42	< 0.001	0.37	< 0.001
ICPV-30m day 1	0.56	< 0.001	0.35	< 0.001
ICPV-4h day 1	0.46	< 0.001	0.28	< 0.001
ICP > 20 day 1	NA	NA	0.60	< 0.001

The table demonstrates the association between the three ICPV measures on day 1 post-injury and proportion of good monitoring time ICP > 20 mmHg on the same day and the following 4 days. However, good monitoring time of ICP > 20 mmHg on day 1 had a stronger correlation with ICP > 20 mmHg day 2 to 5 than the three ICPV measures

*NA* not applicable

The effects of DC on ICPV was evaluated as the temporal trend the first 10 days post-injury, for patients treated with DC in relation to the patients that did not require DC treatment (Fig. 4). Furthermore, the immediate effects of DC were evaluated as the difference in ICPV before and after secondary DC.

p values < 0.05 were considered statistically significant.

### Ethics

All procedures performed in the studies were in accordance with the ethical standards of the institutional and national research committee and with the 1964 Helsinki declaration and its later amendments or comparable ethical standards. Informed consent was obtained from all individual patients included in the study or their next of kin.

## Results

### Demographic and outcome data

Three hundred sixty-two patients were included. Mean age was 47 (± 19) and 79% were male. Eight percent were GCS M 1–2 at admission, 20% had pupillary abnormalities (anisocoria and/or one/two unreactive pupils) and 66% had CT Marshall score diffuse injury I-III. Forty-six percent were operated with craniotomy, 11% treated with thiopental and 10% with DC. Fifty-six percent of the patients had favorable clinical outcome at 6 months following injury.

### Description of ICP variability

**Fig. 2:**
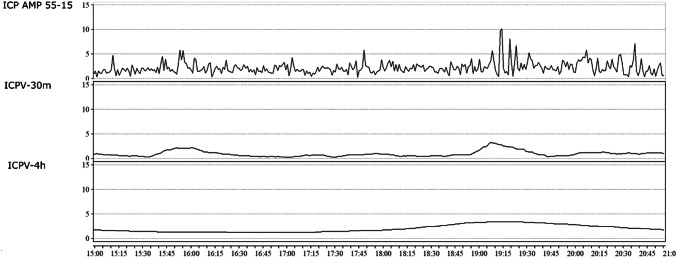
ICP variability in one TBI patient. The figure demonstrates the ICP AMP 55–15, ICPV-30m and ICPV-4h curves during 6 h for one TBI patient. The temporal variation was higher for the short-term variability measure ICP AMP 55–15, whereas it was lower for the long-term variability measures ICPV-30m and even lower with ICPV-4h

**Fig. 3:**
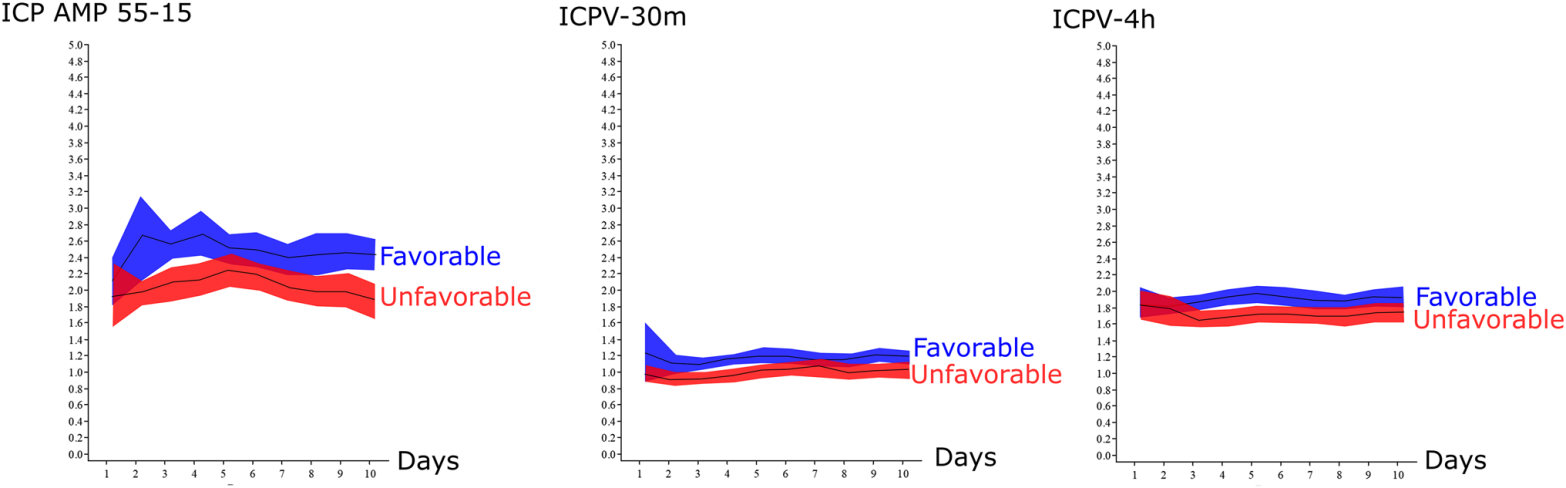
Temporal trends in ICP variability—relation to favorable and unfavorable outcome. Favorable/unfavorable (blue/red) outcome was defined as GOS-E 5–8/1–4. The temporal differences between the outcome groups were most pronounced day 2 to 5. The shaded areas indicate the 95% confidence interval

Figure 2 illustrates a typical example of the differences in temporal variation among ICP AMP 55–15, ICPV-30m and ICPV-4h in one TBI patient. All three ICPV measured mostly varied in the 0–5 mmHg-range (Fig. 3). The ICP AMP 55–15 curve had greater variability in comparison to ICPV-30m and ICPV-4h (Fig. 2).The number of patients with ICP-monitoring data varied from 176 to 310 (49 to 86%) on each of the first 10 days.

### ICP variability: explanatory variables

In the Spearman’s rank correlation analyses, both higher ICPV-30m and ICPV-4h were associated with younger age, whereas ICP AMP 55–15 had no correlation with age (Table 1). Higher ICPV values for all three measures were associated with higher GCS M/better neurological status at admission and lower CT Marshall score.

Higher ICPV for all three measures was also associated with a reduced intracranial reserve, defined as high ICP and RAP, respectively. Furthermore, higher ICP AMP 55–15 was associated with higher ART AMP 55–15. Similarly, ICPV-30m and ICPV-4h correlated positively with BPV-30m and BPV-4h, respectively.

All these demographic and physiologic variables were included in three multiple linear regression analyses to predict each of the three ICPV measures (Table 1). Higher age was associated with a reduced ICP AMP 55–15 and ICPV-4h, but not ICPV-30m. Normal pupillary status was associated with higher ICPV-30m, but not ICP AMP 55–15 and ICPV-4h. High ICP and RAP were significantly associated with higher ICPV for all time intervals. Blood pressure variability (ART AMP 55–15, BPV-30m and BPV-4h, respectively) had a positive correlation with the corresponding time interval for ICP variability.

### ICP variability: outcome prediction

The temporal course for the three ICPV measures the first 10 days post-injury for those with favorable and unfavorable outcome is illustrated in Fig. 3. All ICPV measures were significantly higher for those with favorable outcome, particularly day 2 to 5. In this time interval, mean ICPV values for favorable and unfavorable outcome were 2.5 ± 1.0 vs. 2.2 ± 1.4 mmHg (*p* value = 0.003) in ICP AMP 55–15, 1.2 ± 0.5 vs. 0.9 ± 0.4 mmHg (p value < 0.001) in ICPV-30m and 1.9 ± 0.5 vs. 1.7 ± 0.6 mmHg (p value = 0.006) in ICPV-4h. Lower ICPV was also associated with unfavorable outcome in a simple logistic regression analysis (Table 2).

Three multiple logistic regression analyses were done to evaluate if each ICP variability measure carried additional important information for outcome prediction after adjustment for age, GCS M and pupillary status at admission, DC (yes/no), GMT  % ICP > 20 mmHg, GMT % 70 mmHg > CPP > 60 mmHg, PRx55–15 and blood pressure variability (ART AMP 55–15, BPV-30m or BPV-4h for the corresponding time interval for ICP variability, respectively (Table 3). Higher age, lower GCS M, presence of pupillary abnormalities and higher PRx55–15 were significant predictors for poor clinical outcome in all three regressions. Furthermore, higher ICP AMP 55–15 and ICPV-30m independently predicted favorable outcome, whereas ICPV-4h was not associated with outcome. Neither GMT  % ICP > 20 mmHg nor GMT  % 70 mmHg > CPP > 60 mmHg were associated with outcome.

Lower RAP was associated with unfavorable outcome in a simple logistic regression (odds ratio = 0.09, p value < 0.001) and inclusion of RAP in the multiple logistic regression of unfavorable outcome attenuated the association between outcome with ICP AMP 55–15/ICPV-30m and RAP, respectively, to non-significant.

### ICP variability: prediction of intracranial hypertension

All three ICPV measures on day 1 post-injury were strongly associated with ICP > 20 mmHg on the same day (Table 3). Higher ICPV on day 1 also predicted ICP insults on the following 4 days. However, GMT (%) ICP > 20 mmHg day 1, as compared to the ICPV measures, had the strongest correlation with GMT (%) ICP > 20 mmHg on day 2 to 5 (r = 0.60, p < 0.001).

### ICP variability: relation to decompressive craniectomy

**Fig. 4:**
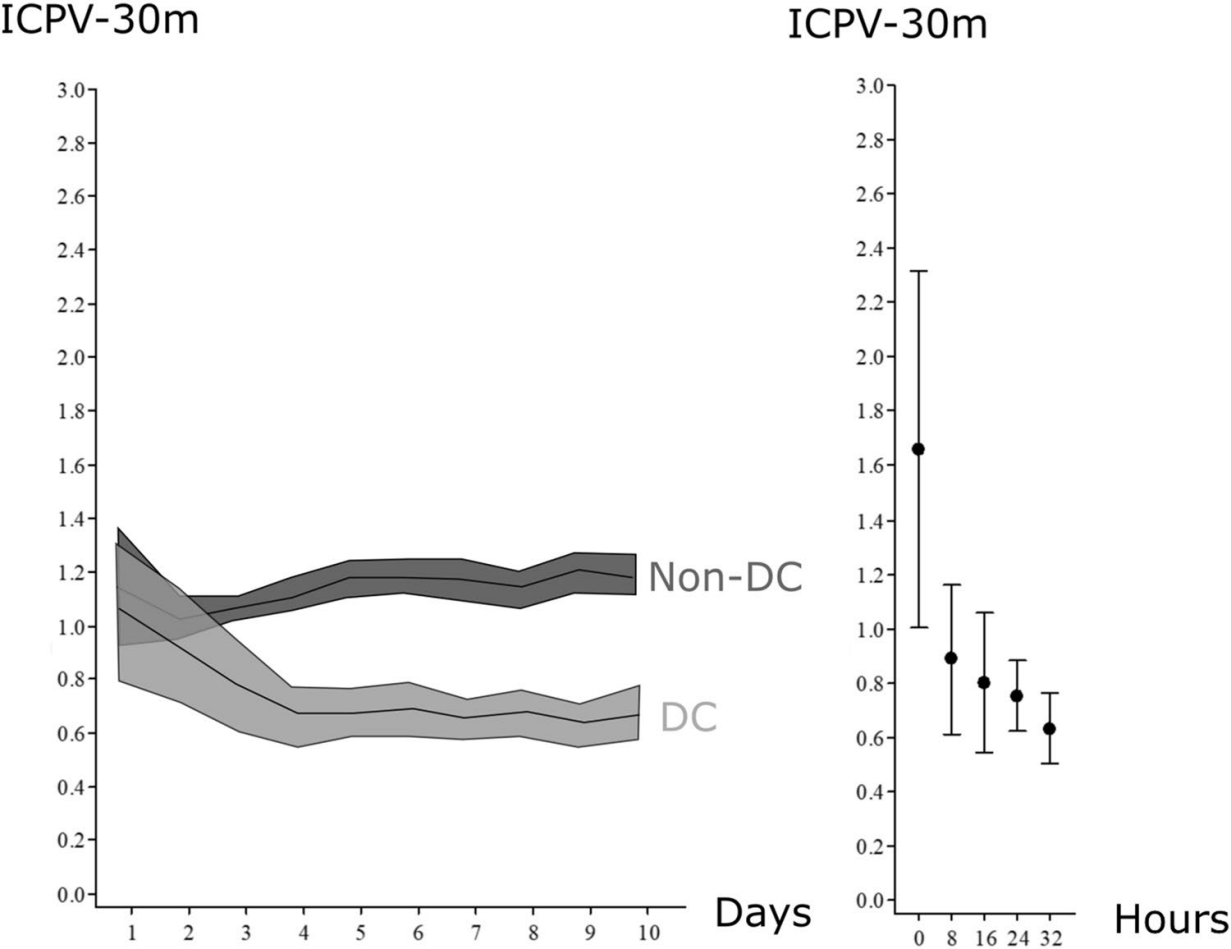
Temporal trends in ICP variability following TBI—DC and non-DC population. The figure on the left demonstrates the temporal courses in ICPV-30m for TBI patients treated with DC and the non-DC population the first 10 days post-injury. The shaded area indicates the 95% confidence interval. The figure on the right demonstrates the effect of DC on ICPV-30m for patients with ICP-monitoring before the surgery. The temporal course includes 8 h-intervals and starts 8 h (at the time point 0) before the DC

The temporal course for ICPV-30m for patients treated with DC (n = 37) and the non-DC (n = 325) population is illustrated in Fig. 4. ICPV was significantly higher for the non-DC population, whereas the ICPV in the DC group gradually decreased. DC surgery was also independently associated with lower ICPV in the multiple linear regression analyses (Table 1). The immediate effect of secondary DC on ICPV is illustrated in Fig. 4. For the measures ICP AMP 55–15 and ICPV-4h, there were similar trends as ICPV-30m in relation to DC (not shown).

## Discussion

In this study, we found that ICPV, particularly high ICP AMP 55–15 and ICPV-30m, independently predicted favorable outcome. The ICPV measures had amplitudes around 0–5 mmHg and likely represented smaller changes in the cerebral blood volume, rather than small changes in mass lesion size or edema. Although higher ICPV was associated with negative factors such as intracranial hypertension and higher blood pressure variability, it also correlated with predictors for better outcome such as younger age, higher GCS M at admission and lower PRx/intact pressure reactivity. This indicates that ICPV is augmented by a reduced intracranial pressure/volume reserve and varies with the cerebral blood volume as a consequence of the vessel response to variable blood pressure. Thus, ICPV may via several mechanisms be associated with better cerebral blood flow regulation and less secondary insults.

### ICPV: explanatory variables

ICP variations have generated interest since the development of ICP monitoring in the NIC [17]. Various ICP wave forms have been examined such as Lundberg’s type A wave (plateau wave) with amplitudes above 50 mmHg, a duration above 5 min and occurring at a relatively low frequency [18]. Type B waves have a higher frequency at approximately 1–2 waves/min and with a lower amplitude around 5 mmHg. Recently, ICP variability has also been examined with mathematical tools using e.g. frequency domain approaches, confining the ICP analysis to variations within specified slow-wave ICP frequencies [18].

The origin of ICPV has been debated [17]. Rosner suggested that both type A and B waves originated from the same mechanisms, i.e. a vessel reaction due to unstable blood pressure, generating variations in cerebral blood volume and ICP [19]. These reactions are amplified when the intracranial pressure/volume reserve is reduced. Other explanations are related to cerebral blood flow-metabolism, variations in arterial pCO_2_ and rhythmic brainstem oscillations that control the cerebral vessels [17, 18, 20].

In line with these theories, we found that reduced intracranial volume/pressure reserve (high ICP and RAP) and high BPV (ART AMP 55–15/BPV-30m/BPV-4h) predicted high ICPV in the multiple linear regressions (Table 1). The univariate analysis showed that ICPV was associated with intact pressure reactivity (low PRx), indicating that the patients with high ICPV generally had healthy, responsive cerebral vessels. On the other hand, PRx was not a significant predictor of high ICPV in the multiple model, indicating that the relationship is not causal. This is consistent with the fact that ICPV was strongly associated with elevated ICP and reduced intracranial compliance (high RAP), probably caused by cerebrovascular dilation and increased cerebral blood volume (CBV). With high ICP, pressure reactivity has the opposite effect of triggering vascular contraction, decreasing ICP and increasing intracranial compliance. Because of the association of ICPV with favorable outcome it is unlikely that the associated increases in CBV are due to the pathological, passive vasodilation associated with hyperperfusion, hyperemia and extreme, untreatable ICP. Instead this appears to be a controlled vasodilation, probably in response to metabolic demand, with net positive effects. This conclusion could be compared to Czosnyka et al. who found that higher RAP, similar to ICPV, was associated with better clinical outcome and reflected the upper limit of vasodilatory autoregulation [21]. It is also likely that higher ICPV reflected cerebral vessels that were healthier in terms of more compliant and less stiff, enabling greater flexibility in cerebral blood volume and ICP in response to blood pressure variability. It would be very interesting to further evaluate the relation between ICPV, CBF and cerebral energy metabolism in future studies by means of other modalities such as transcranial Doppler, brain tissue oxygen monitors and cerebral microdialys.

Furthermore, normal pupillary status at admission independently predicted higher ICPV. As pupillary abnormalities could be related to brain herniation and brainstem injuries, the rhythmic brainstem ICP oscillations may have become deranged, leading to a reduced ICPV [20].

Decompressive craniectomy (DC) independently predicted low ICPV for all time intervals. DC drastically changes the intracranial dynamics as the cranial vault is opened, with an increased intracranial pressure/volume reserve [13]. Hence, variations in intracranial volume would generate only small ICPV values post-DC.

**Fig. 5:**
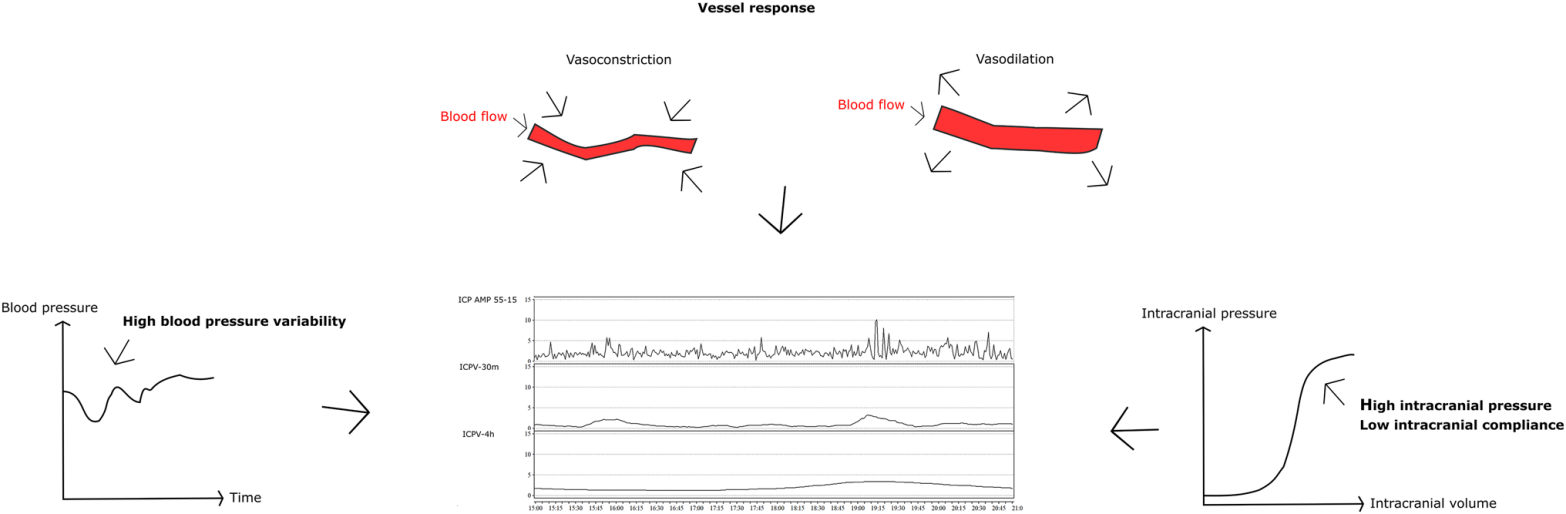
ICP variability—explanatory variables. Schematic drawing of the main explanatory variables for ICPV. ICPV is caused by temporal changes in cerebral blood volume due to variations in blood pressure (left) and the cerebral vessel response (top). These changes in cerebral blood volume are amplified by a reduced intracranial pressure/volume reserve (right)

Castellani et al. looked at ICPV in terms of plateau waves in severe TBI. Similar to our findings, high ICPV/plateau waves were associated with young age, diffuse rather than focal brain injury, high ICP/RAP and intact pressure autoregulation [22]. We believe, that high ICPV is explained by a reduced intracranial pressure/volume reserve and the vessel response to variable blood pressure (Fig. 5), which is similar to the conclusions made by Castellani et al. regarding plateau waves.

### ICPV: different time intervals and relation to outcome

Traditionally, ICPV in terms of type A and B waves have been considered pathological [18, 23]. However, we and others have earlier demonstrated that high ICPV is associated with survival and favorable outcome [3–5]. Balestreri et al. defined ICPV as the slow wave amplitudes of ICP oscillations 0.05 to 0.0055 Hz and found higher values for those with favorable in contrast to fatal outcome in a univariate analysis [3]. Kirkness et al. defined ICPV as the root mean square successive difference between 5-s averages and for 5-m, 60-m and 24-h [4]. Logistic regression analyses showed that high averages of the 5-m and 60-m variability measures had the best prediction of survival and favorable outcome. We have also found that ICPV defined as the absolute deviation from the mean value for 4 h was significantly higher for those with favorable compared to unfavorable outcome [5].

In this study, we evaluated three ICPV measures, i.e. very short-term (ICP AMP 55–15), short-term (ICPV-30m) and long-term (ICPV-4h). For all three measures, higher values were associated with better outcome (higher GOS-E). However, only ICP AMP 55–15 and ICPV-30m independently predicted favorable outcome in the multiple logistic regression analysis (Table 3). This indicates that ICPV brings additional physiological information that is important for predicting outcome. However, this relation was attenuated by adding RAP to the multiple logistic regression of outcome, indicating that high RAP and high ICPV-30m represent similar underlying physiological benefits, probably mediated by controlled cerebral vasodilation, as argued above.

A previous study found that the pressure reactivity index was best evaluated in the frequency range with oscillations with periods from 55 to 15 s (PRx55–15) [5, 12]. Similarly, ICP AMP 55–15 was an independent predictor of outcome, but ICPV-30m was a slightly stronger outcome predictor (Table 3). Furthermore, PRx, but not the short-term PRx55–15, was correlated with ICPV in the univariate analysis. These results may indicate that whereas pressure reactivity is a relatively simple reflex, the vascular activities driving significant changes in CBF are more complex and require greater coordination over longer time periods.

### ICPV: prediction of intracranial hypertension

As ICPV is associated with the intracranial pressure/volume reserve and possibly the autoregulatory status [3, 17, 18], we investigated if ICPV would be a valuable tool to predict intracranial hypertension. However, although all ICPV measures correlated strongly with ICP insults, the GMT of ICP insults above 20 mmHg on day 1 had an even stronger association with ICP insults on day 2 to 5 post-injury (Table 2). Hence, ICPV does not bring any additional value to mean ICP for prediction of intracranial hypertension. These conclusions are similar to Balestreri et al., who found that ICPV rather signified than predicted intracranial hypertension [3].

## Conclusions

ICP variability was traditionally considered pathological in acute brain injuries. Contrary to earlier belief, we found that higher ICP variability in the 0–5 mmHg range in the subminute and 30-minute interval independently predicted favorable outcome in traumatic brain injury.

Although higher ICPV was associated with higher intracranial hypertension and higher blood pressure variability, it also correlated with positive factors such as young age and high GCS M at admission. Our analysis led to the conclusion that the intracranial effects and the effect on outcome are both due to a controlled dilation of the cerebral vessels, probably in response to metabolic demand, with net positive effects.
